# Prognostic Significance of Complications after Laparoscopic Colectomy for Colon Cancer

**DOI:** 10.1371/journal.pone.0108348

**Published:** 2014-10-09

**Authors:** Xiang Xia, Weidong Wu, Kundong Zhang, Gang Cen, Tao Jiang, Jun Cao, Kejian Huang, Chen Huang, Zhengjun Qiu

**Affiliations:** Department of General Surgery, Shanghai Jiaotong University Affiliated First People's Hospital, Shanghai, People's Republic of China; Osaka Medical Center for Cancer and Cardiovascular Diseases, Japan

## Abstract

**Aims:**

This study sought to evaluate the prognostic significance of postoperative complications for colon cancer patients undergoing laparoscopic surgery.

**Methods:**

From May 2006 to May 2009, a total 224 patients who underwent laparoscopic curative resection (R0) for colon cancer were included in our retrospective study. Postoperative complications were evaluated according to a standardized grading system. The main outcome measurements of our study were overall survival (OS) and relapse-free survival (RFS), which were then compared between the no complication and complication groups. Univariate and multivariate analysis were used to assess the correlation between complications and prognosis.

**Results:**

Fifty-nine postoperative complications occurred in 43 patients. The overall morbidity rate was 26.3%. The 5-year OS in the complication group was 41.4% compared with 78.5% in the no complication group (P<0.001). The cumulative incidence of relapse was also more aggressive in patients with complications (5-year RFS: complication group 40.9% vs. no complication group 82.1%, P<0.001). Multivariate analysis identified complications as a significant factor increasing the risk for both OS (RR 2.737; 95% CI 1.512–4.952; P = 0.001) and RFS (RR 4.247; 95% CI 2.291–7.876; P<0.001).

**Conclusion:**

Postoperative complications could pose a significant adverse impact not only on OS but also on RFS in patients with colon cancer even when laparoscopic R0 resection is available.

## Background

Colon cancer is one of the most frequent malignant tumors of the digestive tract, with high rates of morbidity and mortality worldwide [1]. Moreover, the incidence rate of colon cancer continues to rise as people change their lifestyles and food habits [2]. Although surgical management remains the mainstay treatment, the laparoscopic approach has recently been promoted as a therapeutic alternative for the treatment of colon disease [3], [4]. In our previous study, we showed that laparoscopic colectomy showed more benefits in terms of postoperative recovery compared to open surgery [5]. In addition, previously published studies on gastrointestinal tumors, such as gastric and esophageal cancers, revealed that postoperative complications such as anastomotic leak could have a significant adverse impact on prognosis [6], [7], [8]. However, few such reports have related to colon cancer, and even fewer have evaluated laparoscopic surgery. Two recent studies demonstrated that anastomotic leak was a major independent prognostic factor for long-term survival in colorectal cancer patients treated with open resection, as the prolonged inflammatory response to anastomotic leak could promote metastasis [9], [10]. Furthermore, we hypothesized that overall postoperative complications, which could lead to a systemic inflammatory response, may also have an effect on prognosis.

In regards to complications, a uniform and standard grading system for postoperative complication has great clinical and research importance. However, the criteria used to evaluate such postoperative complications have varied among previously reported trials. Thus, for dependable quality assessment, relevant data for postoperative outcomes should be obtained in a standardized and reproducible way to allow for comparisons among different medical centers worldwide [11], [12]. In 2004, Dindo et al. [12] proposed a grading system for complications associated with all surgical procedures, which was based on severity as reflected by the therapeutic method. Subsequently, Dindo and coworkers presented reliable evidence concerning the application of this system for surgical complications in a number of fields [13]. Widespread acceptance of the Dindo complication classification system could help to standardize outcome reporting for various surgical arms, including laparoscopic colectomy, thus promoting analyses and comparisons among different hospitals or teams.

In this study, we aimed to investigate the prognostic role of postoperative complications that were stratified using the Dindo grading system in colon cancer patients after laparoscopic surgery.

## Patients and Methods

### Ethics statement

This retrospective study was approved by the Clinical Trial Ethics Committee of Shanghai Jiaotong University Affiliated First People's Hospital. Written consents were obtained for all patients enrolled. The procedures also followed the tenets of the Declaration of Helsinki.

### Patient selection

From May 2006 to May 2009, 224 consecutive patients with colon cancer who underwent elective laparoscopic curative resection(R0) in the Shanghai Jiaotong University Affiliated First People's Hospital were retrospectively recruited from our database. Information including patient characteristics, surgical records, pathological results, postoperative complications and follow-up were obtained from our medical database. Laparoscopic surgery was performed by a stable surgical team, and patients were assigned to laparoscopic surgery according to their target dates for treatment. All patients enrolled received preoperative laboratory examinations including tumor marker screening, coagulation tests, chest x-ray, abdominal ultrasound, colonoscopy, and if necessary, computed tomography (CT) scans of the abdomen and pelvis. All patients were confirmed to have a malignant tumor after postoperative pathological examination. None of the patients had accepted preoperative radiotherapy or chemotherapy.

The exclusion criteria for our study consisted of synchronous metastasis, emergency presentation, conversion to open surgery because of any adverse events, missing any necessary data(such as follow-up), or any combined resection.

### Surgical procedure

Laparoscopic colectomy was performed as a laparoscopic-assisted procedure, with removal of the tumor specimen via a horizontal minilaparotomy (5 cm) just above the mons pubis. Laparoscopic surgery was conducted using a 5-trocar technique with a trocar (10 mm) inserted via a paraumbilical incision (camera port). Four additional (5 mm) trocars were inserted in the left and right lower abdomen. After removal of the resected specimen and preparation of the stapler anastomosis, we closed the minilaparotomy and reintroduced the pneumoperitoneum.

### Evaluation of postoperative complications

Surgical complications were defined as Grades I, II, III, IV and V recommended by Dindo et al [12]. Grade I included any deviation from the normal postoperative course, including wound infection and dehiscence without surgical intervention. Grade II included pharmacological treatment, including parenteral alimentation, transfusion, infection, etc. Grade III included complications requiring surgical or endoscopic intervention, such as wound dehiscence necessitating stitches, aeropleura, anastomotic leak necessitating reoperation, etc. Grade IV included serious complications requiring intensive care unit management and Grade V was associated with postoperative death. Only those complications occurring within one month after surgery were considered. Anastomotic leak was defined as discharge of colon contents via the drain, wound, or abnormal orifice or as diagnosed by CT scan or surgery. Pyrexia of unknown origin was defined as any body temperature greater than 37°C for more than 24 h, which occurred after the original pyrexia following surgery had settled and for which no obvious cause could be found and antibiotics were necessary. Wound infection was diagnosed according to wound cellulitis or discharge of purulent exudates. Urinary infection was defined as the presence of >10^5^ bacteria/ml in addition to white cells in the urine. Other complication included the following: septicemia, defined as positive blood culture; wound dehiscence, defined as superficial or deep wound breakdown; respiratory failure, defined as respiratory difficulty requiring emergencyventilation; andileus, defined as the need for a nasogastric tube for postoperative nausea, vomiting and abdominal distention or delayed oral intake for more than five days after surgery. According to the grading system outlined above, our surgical team divided the224 patients into 2 groups: the no complication group(complication of less than grade II) and the complication group(complication grade II or higher). If 2 or more complications occurred in one patient, the higher grade was used for this analysis. Identical criteria were used for each patient, including preoperative preparation, postoperative management and hospital discharge.

### Follow-up

All patients were followed after discharge from the hospital according to a pre-established protocol. This evaluation, which was performed every 3 months, included a medical history, physical examination, lab tests such as carcinoembryonic antigen (CEA) and carbohydrate antigen (CA) 19-9 levels, survival status, cause of death(cancer-related or not), local tumor recurrence, and distant metastases. Either ultrasonography or CT scan of the abdomen, in addition to colonoscopy, was performed every year for the first 5 years. When colonoscopy was not available, the barium enema method was used. A detailed description of the follow-up program has already been published [5]. For some patients without available data in the clinic, follow-up was obtained through telephone inquiries. The primary outcome measurements of the study were overall survival (OS) and relapse-free survival (RFS). OS was defined as the time of surgery until death or the last follow-up contact. RFS was defined as the time of surgery until the date of suspected tumor recurrence in patients with eventually confirmed tumor recurrence or the last follow-up contact in patients without recurrence. Of the patients who were pathologically diagnosed as stage III, all received adjuvant chemotherapy with oxaliplatin and 5-fluorouracil for 6 months postoperatively.

### Statistics

RFS and OS were evaluated using the Kaplan-Meier method and compared with the log-rank test. Analysis of predictive factors of survival was performed. Variables analyzed univariately were age, gender, ASA(American Society of Anesthesiologists) scores, body mass index (BMI), tumor location, preoperative comorbidities, duration of operation, tumor maximum size, operation type, estimated blood loss, UICC(International Union Against Cancer) stage and postoperative complications. Variables significantly associated with RFS or OS were then used for multivariate analysis using a stepwise Cox proportional-hazards regression model. Statistical significance was defined as P<0.05. All calculations were performed using the SPSS software package version 13.0 (SPSS Inc., Chicago, IL).

## Results

At the time of final follow-up (April 23rd, 2014), fifty-nine patients (26.3%) had died. The median follow-up was 60 (range 6–80) months. A follow-up rate of 100% was achieved in the present study, and all subjects were compliant with the proposed postoperative surveillance protocol.

### Complication overview

**Figure 1:**
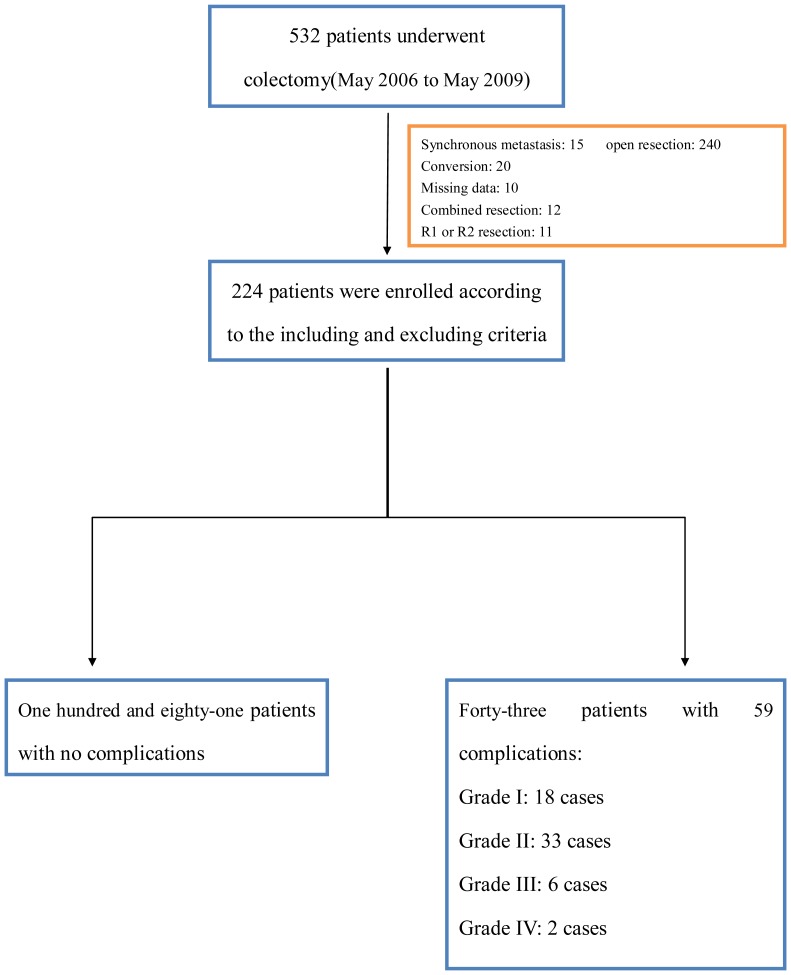
Study flow.

A total of 224 patients were enrolled in this study (Figure 1). Of these, 181 patients were discharged after a smooth recovery without any complications. Fifty-nine postoperative complications occurred in 43 patients, including cases in which two or more morbidities occurred in one patient. Thus, the number of postoperative complications was not equal to the number of patients. The distribution of complications is depicted in Table 1. The overall morbidity rate was 26.3%. Grade I complications(comprising wound infection and wound dehiscence) were recorded in 8.0% of patients. Grade IIcomplications(comprising mainly anatomic leak(treated conservatively), pyrexia of unknown origin, and urinary infection) were recorded in 14.7% of patients. Grade III complications(comprising mainly wound dehiscence(necessitating stitch), anastomotic leak(necessitating reoperation) and intraperitoneal hemorrhage(necessitating reoperation)) were recorded in 2.7% of patients. Grade IV complications were recorded in 0.9% of patients. There were no postoperative deaths in our study.

**Table 1:** Elaboration of Postoperative Complications.

	Number	
Overall postoperative complications	59	26.3%
Grade I	18	8.0%
Wound infection	14	
Wound dehiscence	4	
Grade II	33	14.7%
pyrexia of unknown origin	3	
urinary infection	2	
septicaemia	2	
postoperative hypertension	2	
anastomotic leak(treated conservatively)	3	
Ileus(treated conservatively)	8	
chyle leakage	1	
deep vein thrombosis	2	
intraperitoneal hemorrhage(necessitating transfusion)	4	
respiratory infection	6	
Grade III	6	2.7%
wound dehiscence(necessitating stitch)	2	
anastomotic leak(necessitating reoperation)	2	
intraperitoneal hemorrhage(necessitating reoperation)	2	
Grade IV	2	0.9%
respiratory failure	1	
cardiac failure	1	

The definition of complications had been given in the article.

### Prognostic factors for OS and RFS

The relationship between clinicopathological characteristics and OS in colon cancer patients is shown in Table 2 and Table 3. On univariate analysis, ASA scores, the duration of operation, tumor maximum diameter, UICC stage and complication grade II or higher were significantly associated with OS. Variables that significantly affected OS were selected in the proportional hazards models. Only a duration of operation >150 min(RR 1.919, 95% CI 1.089–3.380, P = 0.024), tumor maximum diameter >5 cm(RR 2.513, 95% CI 1.454–4.343, P = 0.001), UICC stage(II, RR 3.267, 95% CI 1.057–10.096, P = 0.040; III, RR 11.616, 95% CI 4.085–33.032, P<0.001) and complication grade II or higher(RR 2.737, 95% CI 1.512–4.952, P = 0.001) remained independently associated with OS. With regard to RFS, a tumor maximum diameter >5 cm(RR 2.495, 95% CI 1.374–4.531, P = 0.003), UICC stage(II, RR 3.244, 95% CI 1.065–9.879, P = 0.038; III, RR 8.817, 95% CI 3.011–25.817, P<0.001) and complication grade II or higher(RR 4.247, 95% CI 2.291–7.876, P<0.001) were found to be independent prognostic factors for those patients.

**Table 2:** Univariate analysis of prognosis factor for OS and RFS (n = 224).

Characteristic	Category	No of patients	OS		RFS	
			5-year (%)	P	5-year (%)	P
Age				0.162		0.165
	<75	132	77.3		80.8	
	≥75	92	68.5		71.8	
Sex				0.264		0.289
	Male	108	76.9		79.9	
	Female	116	70.7		74.3	
ASA				0.020		0.003
	I–II	170	77.6		82.3	
	III–IV	54	61.1		64.3	
BMI(kg/m^2^)				0.169		0.477
	≤25	168	76.2		77.9	
	>25	56	66.1		74.6	
Tumor Location				0.468		0.343
	Right	84	71.4		73.9	
	Transverse	12	66.7		66.7	
	Left	128	84.4		79.9	
Co-morbidities				0.502		0.075
	Yes	74	75.7		84.8	
	No	150	72.7		73.3	
Operation type(hemicolectomy)				0.265		0.203
	Right	96	70.8		73.1	
	Left	128	75.8		79.9	
During of operation (min)				0.004		0.070
	≤150	162	78.4		79.8	
	>150	62	61.3		69.6	
Tumor maximum diameter (cm)				0.003		0.001
	≤5	150	79.3		83.5	
	>5	74	62.2		63.7	
Estimated blood loss (ml)				0.561		0.888
	≤100	196	74.5		76.9	
	>100	28	67.9		77.5	
UICC				<0.001		<0.001
	I	52	92.0		92.3	
	II	88	83.0		81.7	
	IIA	3	66.6		66.6	
	IIB	67	88.1		86.5	
	IIC	18	66.7		66.7	
	III	84	52.4		68.0	
	IIIA	34	61.7		79.4	
	IIIB	30	50.0		50.0	
	IIIC	20	40.0		49.6	
Complication grade II or higher				<0.001		<0.001
	Yes	29	41.4		40.9	
	No	195	78.5		82.1	

OS overall survival; ASA American society of anesthesiologists; RFS relapse free survival; UICC International Union Against Cancer.

**Table 3:** Multivariate analysis of prognosis factors for OS and RFS.

Covariate	OS			RFS		
	RR	95% CI	P	RR	95% CI	P
During of operation (min)						-
≤150	1	-		-	-	
>150	1.919	1.089–3.380	0.024	-	-	
Tumor maximum diameter (cm)						
≤5	1	-		1	-	
>5	2.513	1.454–4.343	0.001	2.495	1.374–4.531	0.003
UICC						
I	1	-		1	-	
II	3.267	1.057–10.096	0.040	3.244	1.065–9.879	0.038
III	11.616	4.085–33.032	<0.001	8.817	3.011–25.817	<0.001
Complication grade II or higher						
No	1	-		1	-	
Yes	2.737	1.512–4.952	0.001	4.247	2.291–7.876	<0.001

OS overall survival; RFS relapse free survival.

### Complication effects on OS and RFS

**Figure 2:**
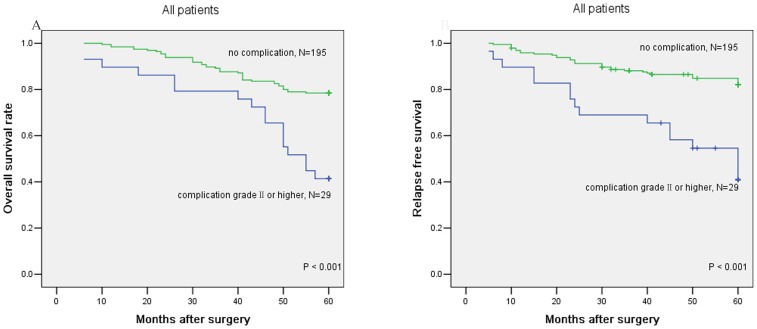
Prognostic significance of complications grade II or higher in the whole population. A: overall survival, B: relapse-free survival. OS and RFS were evaluated using the Kaplan-Meier method and compared with the log-rank test.

**Figure 3:**
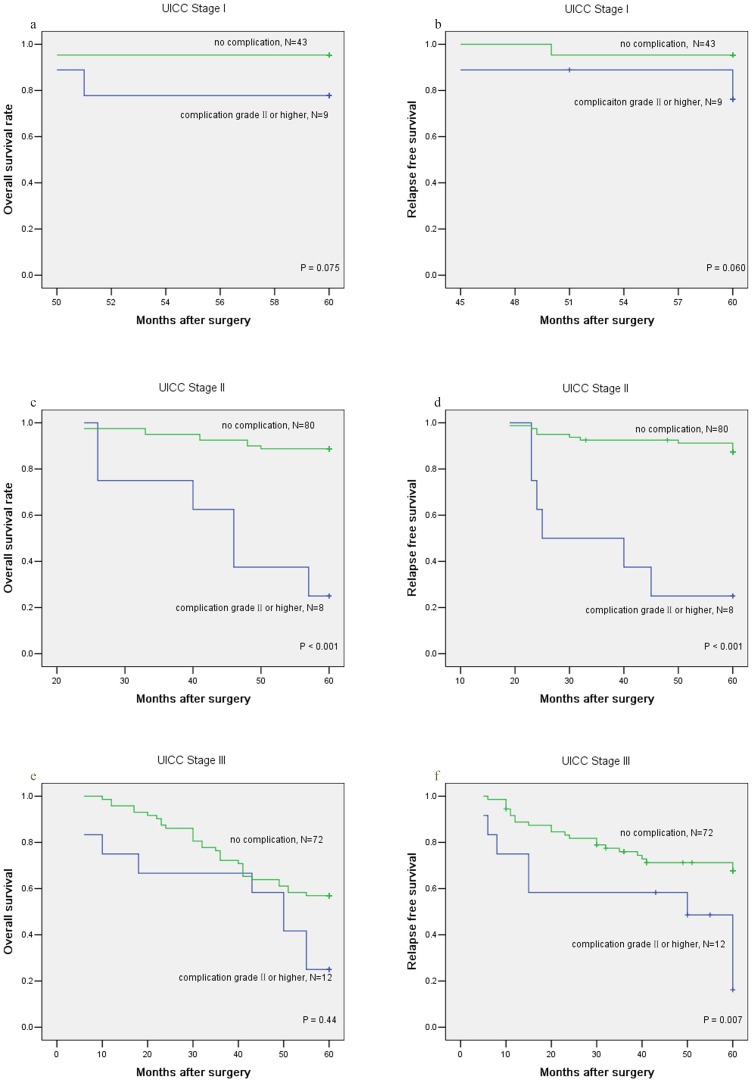
Prognostic significance of complications grade II or higher in the subgroups that were stratified by UICC staging. a, c, e: overall survival; b, d, f: relapse-free survival. OS and RFS were evaluated using the Kaplan-Meier method and compared with the log-rank test.

The OS and RFS results associated with complications are presented graphically in Figure 2A and B, respectively, and the resultant curves stratified by UICC stage are shown in Figure 3. The 5-year OS rate for patients with complication of grade II or higher was 41.4%, which was significantly lower than for those without complications (78.5%, P<0.001) (Table 2, Figure 2A). A similar result was observed for the 5-year RFS rate between these two groups (no complication 82.1% vs. complication grade II or higher 40.9%, P<0.001, Table 2, Figure 2B). In the subgroup analysis, the pattern remained the same as UICC stage II and III progressed (Figure 3).

## Discussion

Many previous studies have shown a correlation between anastomotic leak and an increased risk of relapse and worse prognosis in patients with gastric cancer, colorectal cancer and esophageal cancer [6], [9], [10], [14], [15]. However, the imprecise terminology used to define anastomotic leak may vary from a minor radiologic leak without consequence (grade I) to a leak treated conservatively by antibiotics and bed-side opening of wound treatment (grade II), to one requiring stent placement or surgical reintervention (grade III), and to leaks causing life-threatening sepsis (grade IV), or eventually leading to death (grade V). This lack of standardized, precise terminology prevents rigorous conclusions from being made. Thus, in the present study, our surgical team adopted the Dindo classification, a well-defined system for grading severity, to enable objective and precise documentation of postoperative complications. We found that a postoperative complication of grade II or higher was an independent predictor of diminished OS and RFS for patients with colon cancer who underwent laparoscopic curative resection. Furthermore, in subgroup analysis, this effect was significantly observed for stage II and III patients in comparison to stage I patients. Lerut et al. [6] also reported that complications stratified by the Dindo classification appeared to be a useful prognostic indicator of disease-free survival (DFS) for cancers of the esophagus and gastroesophageal junction. In addition, Kubota et al. [8] analyzed 1,395 gastric cancer patients and concluded that complication of grade II or higher had an obvious impact not only on OS but also on DFS, even if the tumor was curatively resected. Both studies mentioned above shared similar results with ours despite the different digestive tract cancers studied.

Complication grade II or higher includes not only infectious complications but also other inflammatory complications that could possibly delay recovery, increase surgical stress and influence the cellular immune response. Many researchers have confirmed that immunoreactions within the body can serve as a prognostic risk factor for cancer. For instance, Paholyuk et al. [16] found that NK cells play an important role in suppressing the growth of stage II colorectal cancer. Shine et al. and DerHagopian et al. further reported that the systematic inflammatory response syndrome (SIRS) caused by leakage might enhance tumor spreading and metastasis [17], [18]. Given that various inflammatory mediators(tumor necrosis factor (TNF)-α, interleukin (IL)-1β and IL-6) and their receptors control cell motility, invasiveness and survival, it seems reasonable that SIRS induced by anastomotic failure may promote proliferation and metastasis in circulating cancer cells and those present in the tumor bed [14], [19], [20], [21]. The notion that an inflammatory response can promote colorectal cancer recurrence and metastasis was further supported by studies of non-gastroenteric tumors, such as breast cancer [22], [23]. In previous work, our surgical team showed that laparoscopic colorectal resection induced less surgical stress and more short-term advantages compared with open resection, as the inflammatory response appeared to be less affected by laparoscopy, according to the counts of CD4 +T cells, CD8 +T cells, CD45RO +T cells and NK cells after surgery [24]. Then, in this study, we found that postoperative complications grade II or higher, which can have a significant effect on the immune system, adversely affected OS and RFS in colon cancer patients.

The mechanism underlying the negative impact of complications on survival remains open to speculation. One of the substantial pieces of evidence supporting the adverse effect of anastomotic leakage on survival is currently presumed to involve the release of exfoliated cancer cells remaining in the bowel lumen [9]. However, the rate of local recurrence (in particular anastomotic recurrence) shows no correlation with the detection rate of exfoliated cells; so, under conditions of local inflammation, the in vivo growth potential of these cells appears to be limited [10]. Thus, it is more likely that prolonged systemic inflammatory responses resulting from complications play an important role because even after curative resection, tumor cells remain and can cause relapse several years later [25]. The inflammatory responses to severe complications are associated with host immunosuppression [19], [26]. Consequently, the residual cancer cells become active enough to develop relapse under those circumstances [6]. Kubota et al. [8] tested postoperative white blood cell counts, C reactive protein (CRP) levels and body temperature and found that these lab data relevant to inflammatory status were significantly higher in the complication group than in the no complication group. Furthermore, our results regarding differences in OS and RFS between patients with and without complications, which were especially remarkable at stage II and III, most likely reflected the potential or the quantity of residual cancer cells. Similar results were also observed in gastric cancer patients [8]. Nevertheless, the relationship between inflammation and poor prognosis remains unclear, and more detailed investigation is clearly required to address this issue. Together, our results indicate that postoperative complications grade II or higher have a negative impact on prognosis. Therefore, these patients should be followed prudently. However, with regard to stage I patients, the difference was not significant (Figure 3 a, b), which may indicate that fewer residual cancer cells in the early stage are not sufficient to cause relapse, even following inflammatory stimulation. In fact, cardiovascular diseases and chronic obstructive pulmonary disease (COPD) were the major causes of deaths in the stage I group(data not shown).

The present study had several limitations. First, conversion patients were excluded from our analysis. However, this study was focused specifically on laparoscopic surgery in colon cancer patients, and there have been several articles about the prognostic analysis for open colon resection. Additionally, selection bias could have been lowered to some extent by excluding conversion patients because of their relatively higher complication rate. Second, although we minimized this selection bias via the inclusion and exclusion criteria, the retrospective nature of our database may have introduced a few inevitable biases. Lastly, our discussion excluded the other two significant covariates: the duration of operation and tumor maximum diameter. In our experience, these two indicators may be a consequence of other factors, such as advanced tumor stage and tumor location. However, further analysis of these two factors will be addressed in our future work.

In conclusion, our study provides the first evidence of complications associated with prognosis after laparoscopic surgery in colon cancer patients, according to a reliable and uniform complication severity grade scale rather than the imprecise terminologies of ‘minor or major complications.’ Complication grade II or higher was found to have a significant adverse impact not only on OS but also on RFS in patients with colon cancer, even when laparoscopic R0 resection was available. Thus, surgeons should work to lower the incidence of complications, and patients with postoperative complications need to be carefully followed for relapse in the first 5 postoperative years.

## Supporting Information

File S1 **All data showed in the manuscript were included in this “plos one upload” file.**
(XLSX)
